# Prognostics and Clinical Outcomes in Patients Diagnosed With Acute Lymphoblastic Leukemia in King Abdulaziz University Hospital, Jeddah, Saudi Arabia

**DOI:** 10.7759/cureus.22952

**Published:** 2022-03-08

**Authors:** Ashwaq T Alghamdi, Joud E Alead, Eman G Darwish, Shahad T Matasif, Mohamad H Qari

**Affiliations:** 1 Cardiology, King Abdulaziz University Faculty of Medicine, Jeddah, SAU; 2 Pediatrics, King Abdulaziz University Faculty of Medicine, Jeddah, SAU; 3 Medicine, King Abdulaziz University Faculty of Medicine, Jeddah, SAU; 4 Hematology, King Abdulaziz University Hospital, Jeddah, SAU

**Keywords:** kingdom of saudi arabia (ksa), white blood count (wbc), clinical outcomes, acute lymphoblastic leukemia (all), prognosis

## Abstract

Background

Acute lymphoblastic leukemia (ALL) is a hematological cancer that causes an accumulation of immature cells in the bone marrow. The count of white blood cells (WBCs) is an independent predictor of survival. Integrating first-line treatment, such as intensive chemotherapy, with prognostic factors aids in developing critical therapeutic decisions and improving long-term outcomes. This study evaluated several prognostics such as age, WBCs, ALL cell subtypes, and absolute WBC counts.

Methods

This study involved a retrospective record review and was conducted by scanning the medical records of all individuals who developed ALL and were on chemotherapy at a teaching Hospital in Jeddah between 2012 and 2018. The data entry was done using Microsoft Excel, while the analysis was done using SPSS Version 21. To test any associations, frequency and measure of central tendencies, t-test, and chi-square test were used.

Results

A total of 98 of ALL patients were on chemotherapy, and 18 were excluded. Thus, 80 patients were analyzed. The mean age for all patients was 13.6 years (range: 0.6-26.6 years), and the most frequent ages were less than 18 years (90%). More than half of them (62.5%) were males. The majority of the patients were Bangladeshi, Pakistani, Indian, Afghan, Indonesian, and Myanmar (37.7%), and the least were Saudi (3.8%). B subtype (75.9%) was more common than T subtype (24.1%). The first remission after treatment was in 66 patients, with a mean of 6.86 years. There was a significant adverse relationship between the ability of patients to reach the first remission and WBC count (p = 0.032). There was strong significant negative correlation between absolute lymphocyte count (ALC) and survival duration after treatment (r = -0.669; p = 0.012).

Conclusions

The impact regarding age and WBC is almost like most previous studies. ALC shows a strong poor prognosis, while ALL cell subtypes demonstrate a contradictory prognosis effect.

## Introduction

Acute lymphoblastic leukemia (ALL) is a bone marrow hematological malignancy that affects lymphoid precursors and results in the accumulation of immature bone marrow blast cells. It has been found in more than 6,500 people per year in the United States alone and is considered the second most common acute leukemia in adults [1,2], as well as the most common childhood malignancy. It is responsible for 75-80% of acute leukemia in children and 20% of acute leukemia in adults [1].

According to the affected lymphoid cell lineage, there are three classifications: B-cell ALL, T-cell ALL, and mixed-lineage ALL [3]. T-cell ALL has a poorer prognosis than B-cell ALL, though childhood T-cell ALL prognoses have improved dramatically. The fact that T-cell ALL cases have different outcomes suggests that this may contribute to chemo-resistance. In recent years, extensive research into T-lymphocyte development has revealed a subset of patients with a phenotype resembling an early T-cell precursor who have a worse prognosis than any other type of T-cell ALL [4].

The number of white blood cells (WBCs) is an independent predictor of survival. At the time of diagnosis, the WBC count appears to be high (>30x109 in B-cell ALL or >100x109 in T-cell ALL) [2]. The new first-line treatment consists of intensive chemotherapy, bone marrow transplantation, and targeted therapy; combining these treatments with prognostic factor analysis aids in developing critical therapeutic decisions, thereby improving long-term outcomes [5].

Intensification of chemotherapy has reduced the risk of relapse in childhood, [6] but the relapse rate in ALL is nearly 15-20%, and the cure opportunity is significantly reduced after relapse. The cause of these relapses is the stability of residual malignant cells, known as minimal residual disease (MRD) [1]. T-cell ALL affects lymphoid-cell-producing stem cells; this subtype accounts for 10%-15% of ALL in children and 25% of ALL in adults. The adoption of intensive chemotherapy improved the prognosis noticeably, and nearly 60% of patients can now be treated [7].

Certain factors, such as abnormal changes in chromosome structure and number, genetic and epigenetic alterations, and absolute lymphocyte count (ALC), that are associated with treatment response and outcome have been identified as contributing to the development of ALL [8,9].

Regarding ALC after induction of chemotherapy, it has been linked to positive presenting characteristics and good early treatment response. However, in recent experiments that treated children with ALL, the prognostic role of ALC in comparison to other prognostics is unknown [9]. The aim of this study was to evaluate several prognostic factors (age, WBCs, ALL-cell types, and ALC) and clinical outcomes in patients diagnosed with ALL and treated with chemotherapy in King Abdulaziz University Hospital (KAUH), Jeddah, Saudi Arabia.

## Materials and methods

Study design and setting

This was a retrospective analysis of records of patients who were diagnosed with ALL by performing bone marrow aspiration and confirmed with flow cytometry to have ALL. A retrospective record review was conducted by scanning the records of all individuals who developed ALL.

Study participants

The study included all ALL patients who were on chemotherapy during the period of 2012 to 2018 at the Department of Hematology, KAUH. Patients were excluded if their data were missing regarding the type of treatment, induction date, or incomplete follow-up. A total of 98 ALL patients who were on chemotherapy were included in this study, and 18 were excluded due to incomplete data; thus, 80 patients were analyzed to explore the prognostics and clinical outcomes.

Data collection

Data were collected regarding chemotherapy induction date, their clinical outcome after treatment including remission and the required duration to reach it, occurrence of relapse, survival, and the survival duration after treatment (SDAT). Baseline age, WBCs count, ALL T-cell and B-cell subtypes, and ALC were prognostic factors.

Ethical considerations

This study was approved by the Unit of Biomedical Ethics - Research Committee at King Abdulaziz University Faculty of Medicine.

Statistical analysis was performed using Statistical Package for Social Sciences (SPSS) Version 21 (IBM Corp., Armonk, NY, USA). For the qualitative and quantitative data, we used frequency and measure of central tendencies respectively. For the bivariate data comparison, we used independent t-test (means) and Pearson’s correlation with normally distributed variables, Mann-Whitney U test (mean ranks) and Spearman’s correlation with nonnormally distributed variables, and chi-square test and Kruskal-Wallis test for multivariate analysis. A p-value of <0.05 was considered significant.

## Results

Population size and percentage

The mean age for all patients was 13.6 years (range: 0.6 -26.6 years), and the most frequent ages were less than 18 years (90%). Out of 80 patients, more than half of the sample was male (62.5%). The clear majority were Asians 37.7%, while Saudis were less (3.8%). Further illustration is in Table 1.

**Table 1:** General description of the population

Variable	Percentage (n = 80)	Duration
Age		
Younger than 18 years	90%	
Older than 18 years	10%	
Gender		
Male	62.5%	
Female	37.5%	
Ethnicity		
Asians	37.5%	
Arabs	53.8%	
Africans	8.7%	
Nationality:		
Bangladeshi	2.5%	
Pakistani	8.75%	
Indian	2.5%	
Afghan	1.25%	
Indonesian	1.25%	
Myanmar	21.25%	
Saudi	3.75%	
Yemeni	32.5%	
Syrian	13.75%	
Palestinian	2.5%	
Jordanian	1.25%	
Somali	3.75%	
Egyptian	1.25%	
Ethiopian	1.25%	
Sudani	2.5%	
Cell subtypes (out of 54)		
B-cell subtype	75.9%	
T-cell subtype	24.1%	
Chronic disease	6.3%	
Initial remission: time required after treatment	82.5%	65 days, IQR = 14.75-59.5
First relapse	64.6%	
Alive at the end of the study	88.1%	
Surviving		
Survived, average of SDAT until the end of the study	80%	1043 days
Died, average of SDAT	20%	417.8 days

Survival duration after treatment and age at diagnosis

Reaching the first remission after treatment was found in 66 patients with a mean of 6.86 years, and there was an adverse relationship between age and the duration elapsed before reaching first remission (r = -0.371; p = 0.002.) At the end of the study, 64 (80%) patients were still alive and the mean age was 7.23 years, (95% CI: 5.2 to 9.27), but the relation was not significant. On other hand, a weak proportional correlation between age and SDAT (r = 0.041; p = 0.884). Death was observed in 16 (20%) patients, and the mean age was 13.8 years (95% CI: 6.39 to 21.24).

ALL subtype 

Of all B-cell subtype patients, 41 (75%) reached the first remission, in addition to 13 (100%) of T-cell subtype patients. Of all 80 patients, 54.7% of B-cell subtypes were alive; furthermore, 20.8% were alive from T-cell subtype.

WBC counts at diagnosis

A significant adverse relationship was found between the ability of patients to reach the first remission and WBC count (p = 0.032). As the count increased, the incidence of remission decreased. There was a weak negative correlation between WBC count and the duration elapsed before accomplishing the remission (r= -0.139), while WBC count had no impact on relapse events (p = 0.833). Moreover, the association between death and WBCs count tended to be higher among patients who died (p = 0.156); mean rank using the Mann-Whitney U test was 47.5 among 16 dead patients vs 38.2 among 61 alive patients. A weak negative correlation was between WBC and SDAT (r = -0.326; p = 0.256). In addition, WBC count distribution among Asians, Arabs, and Africans was not significantly different (p = 0.357).

ALC at diagnosis

There was no correlation between ALC and the duration passed before remission (r = 0.07; p = 0.575). In addition, there was no association between survived and dead patients, and the mean ranks were 39.34 and 40.21, respectively (p = 0.896). However, there was a strong significant negative correlation between ALC and SDAT (r = -0.669; p = 0.012), while ALC was not associated with relapse events.

## Discussion

Investigating multiple prognostics and clinical outcomes at the same time may help in differentiating and evaluating their roles in our population and in general. According to our findings, age is a major useful prognostic indicator for outcomes; when a patient is younger, less than 18 years old, a good prognosis is expected. The first remission was achieved earlier in young patients, which may be attributed to the good condition of the vital organs, the good regeneration of cells, and the absence of any concomitant chronic diseases that may restrain or interrupt recovery. Several studies supported our conclusion that the younger the patients are the more likely they are to have a good prognosis and reach their first remission sooner [10].

This study aimed to find a correlation between WBC count and first remission to evaluate these factors. In our study and previous studies, the majority of our patients have a high WBC count, which indicates a poor prognosis. There was also a low WBC count on the eighth day of induction, which is good as a prognostic factor due to the rapid response of induction and a higher chance of complete remission, and vice versa. We are bolstering our findings with previous research [11].

Furthermore, the finding suggests that the ALC at diagnosis has no effect on the time required to achieve remission and survive, but it has a strong negative association with the SDAT. Although the sample size was small, this suggests that it is a good predictor of survival duration in particular. And several studies [5,9,12] supported this theory regarding different ALC count measuring times, mostly at day 15.

Delaying the time of measuring ALC during induction has been shown in various studies to be a better predictor of treatment response. And this explains why our study, which measured ALC at diagnosis, found no correlation between the duration before remission and the ability to survive when compared to other studies despite the fact that it could be a significant prognostic factor in those aspects with larger studies. Furthermore, B-cell subtype shows better prognosis than T-cell subtype by 24.11% in terms of surviving. This agrees with previous studies [13] that showed T-cell ALL usually demonstrates worse prognosis compared with B-cell ALL. However, the ability to reach the first remission is more occurring with T-cell subtype patients by 33.9%; in addition, no study considered this assumption. Thereby, more research is required to discriminate and explore the outcomes for both types. Furthermore, some data, particularly cell subtypes, were not recorded for all patients, preventing us from conducting the research properly.

In the present study, there are some limitations. First, the study was retrospective in nature and there were incomplete recorded data. Second, missing data within a variable led to a variation in sample between the variables and thus may impact the estimation. Third, this study was restricted to a single center.

## Conclusions

The aim of this study was to evaluate several prognostic factors (age, WBCs, ALL-cell types, and ALC) and clinical outcomes in patients diagnosed with ALL and treated with chemotherapy in KAUH, Jeddah, Saudi Arabia. The impact regarding age and WBC is almost like most previous studies. ALC shows a strong poor prognosis, while ALL cell subtypes demonstrated opposite prognosis effect. T-cell subtype was superior to B-cell subtype in reaching the first remission, while B-cell subtype was superior in survival.
